# Cervical non-invasive vagus nerve stimulation (nVNS) for preventive and acute treatment of episodic and chronic migraine and migraine-associated sleep disturbance: preliminary findings from a prospective observational cohort study

**DOI:** 10.1186/s10194-015-0582-9

**Published:** 2015-12-03

**Authors:** Thomas M. Kinfe, Bogdan Pintea, Sajjad Muhammad, Sebastian Zaremba, Sandra Roeske, Bruce J. Simon, Hartmut Vatter

**Affiliations:** Division of Functional Neurosurgery and Neuromodulation, Department of Neurosurgery, Rheinische Friedrich-Wilhelms University, Regina-Pacis-Weg 3, 53113 Bonn, Germany; Department of Neurosurgery, Rheinische Friedrich-Wilhelms University, Regina-Pacis-Weg 3, 53113 Bonn, Germany; Sleep Medicine, Department of Neurology, Rheinische Friedrich-Wilhelms University, Sigmund-Freud-Str. 25, D-53105 Bonn, Germany; German Centre for Neurodegenerative Diseases (DZNE), Ernst-Robert-Curtius-Str. 12, 53117 Bonn, Germany; electroCore, LLC, 150 Allen Road, Suite 201, Basking Ridge, NJ 07920 USA

**Keywords:** Neuromodulation, Headache, Acute therapy, Prophylactic therapy, Sleep impairment

## Abstract

**Background:**

The debilitating nature of migraine and challenges associated with treatment-refractory migraine have a profound impact on patients. With the need for alternatives to pharmacologic agents, vagus nerve stimulation has demonstrated efficacy in treatment-refractory primary headache disorders. We investigated the use of cervical non-invasive vagus nerve stimulation (nVNS) for the acute treatment and prevention of migraine attacks in treatment-refractory episodic and chronic migraine (EM and CM) and evaluated the impact of nVNS on migraine-associated sleep disturbance, disability, and depressive symptoms.

**Methods:**

Twenty patients with treatment-refractory migraine were enrolled in this 3-month, open-label, prospective observational study. Patients administered nVNS prophylactically twice daily at prespecified times and acutely as adjunctive therapy for migraine attacks. The following parameters were evaluated: pain intensity (visual analogue scale [VAS]); number of headache days per month and number of migraine attacks per month; number of acutely treated attacks; sleep quality (Pittsburgh Sleep Quality Index [PSQI]); migraine disability assessment (MIDAS); depressive symptoms (Beck Depression Inventory® [BDI]); and adverse events (AEs).

**Results:**

Of the 20 enrolled patients, 10 patients each had been diagnosed with EM and CM. Prophylaxis with nVNS was associated with significant overall reductions in patient-perceived pain intensity; median (interquartile range) VAS scores at baseline versus 3 months were 8.0 (7.5, 8.0) versus 4.0 (3.5, 5.0) points (*p* < 0.001). Baseline versus 3-month values (mean ± standard error of the mean) were 14.7 ± 0.9 versus 8.9 ± 0.8 (*p* < 0.001) for the number of headache days per month and 7.3 ± 0.9 versus 4.5 ± 0.6 (*p* < 0.001) for the number of attacks per month. Significant improvements were also noted in MIDAS (*p* < 0.001), BDI (*p* < 0.001), and PSQI global (*p* < 0.001) scores. No severe or serious AEs occurred.

**Conclusion:**

In this study, treatment with nVNS was safe and provided clinically meaningful decreases in the frequency and intensity of migraine attacks in patients with treatment-refractory migraine. Improvements in migraine-associated disability, depression, and sleep quality were also noted.

## Background

As a highly prevalent neurologic disorder, migraine headache exerts a considerable burden on individuals and society [1, 2], including substantial economic costs [3]. Recent findings from the Global Burden of Disease Study 2013 suggest that migraine ranks sixth among the top worldwide causes of disability [4, 5]. Along with premonitory (i.e. aura) and attack-associated symptoms (i.e. phonophobia, photophobia, nausea, and vomiting) [2], patients with migraine are likely to experience sleep disturbances [6, 7] and other comorbidities such as depression and anxiety [8, 9]. Sleep disturbances may trigger a migraine attack in the preictal state [6]. Patients with non-sleep migraine (NSM) demonstrate low thermal pain thresholds, whereas insufficient rest may evoke migraine attacks in patients with sleep migraine (SM) [6, 10].

Although there is no clear consensus on precisely how to define refractory migraine, a key parameter among commonly used clinical definitions is unresponsiveness to medications from multiple pharmacologic classes [11]. Thus, individuals with treatment-refractory migraine require alternatives to standard pharmacologic therapies. Neuromodulation therapy using implanted vagus nerve stimulation (VNS) devices has been successfully used to treat drug-resistant epilepsy [12] and depression [13], and numerous other methods of neuromodulation (e.g. occipital nerve stimulation, non-invasive VNS, transcranial direct current stimulation, repetitive transcranial magnetic stimulation, transcutaneous electrical nerve stimulation, transcutaneous supraorbital nerve stimulation, spinal cord stimulation) have been investigated for treating patients with migraine, with varying degrees of success [14]. Small studies and case reports have shown that implanted VNS may also alleviate migraine and cluster headache [15–18]. Data suggest that attenuation of pain by VNS occurs via inhibition of signalling through afferent vagus nerve fibres to the trigeminal nucleus caudalis (TNC) [19] and via modulation of inhibitory neurotransmitter release, resulting in decreased glutamate levels in the TNC [20, 21].

The use of implanted VNS devices for the treatment of headache disorders is hampered by inherent procedural risks (i.e. infection, lead migration, lead fracture, and battery replacement), health complications (i.e. voice disturbance, cough, headache, and paraesthesia), surgery cost, and the need for postoperative monitoring [22, 23]. Thus, a non-invasive vagus nerve stimulation (nVNS) device (gammaCore®; electroCore, LLC) has been developed and is CE-marked for the treatment of primary headache disorders [24]. Recent evidence suggests that nVNS is effective in the acute treatment of migraine [25, 26] and in the acute or prophylactic treatment of cluster headache [27–29]. An open-label pilot study that evaluated nVNS for the acute treatment of episodic migraine (EM) attacks reported that the efficacy of nVNS at 2 h after initiation of therapy was comparable to that of first-line pharmacologic interventions [25]. Barbanti and colleagues evaluated the use of nVNS for the acute treatment of migraine attacks in patients with chronic migraine (CM) and high-frequency EM [26]. The majority of patients with mild or moderate migraine attacks achieved pain relief or pain-free status at both 1 and 2 h after nVNS treatment [26].

No published studies to date have examined the effect of nVNS on sleep quality and depression in patients with migraine. We therefore conducted a 3-month, open-label, prospective, observational cohort study to investigate the safety and efficacy of acute and prophylactic nVNS treatment in patients with EM and CM and assess the effects of nVNS on sleep quality in these patients.

## Methods

### Study design

This was a 3-month, single-centre, open-label, prospective, observational cohort study to evaluate the impact of preventive and acute treatment with nVNS in patients with treatment-refractory EM and CM and migraine-associated sleep disturbances, disability, and depressive symptoms. Patients were referred to our department by a headache specialist (neurologist), and their diagnoses were confirmed by a multidisciplinary pain board consisting of neurologists, anaesthesiologists, neurosurgeons, psychiatrists, and pain nurses.

### Ethics, consent, and permissions

Approval for this study was obtained from the institutional ethics committee. All patients provided written informed consent.

### Study population

Patients who were diagnosed with EM (headaches occurring <15 days per month) or CM (headaches occurring ≥15 days per month) according to the *International Classification of Headache Disorders* criteria (3rd edition; beta version) [2] and who fulfilled all of the inclusion criteria and none of the exclusion criteria (Table 1) were enrolled. All patients enrolled in the study were considered refractory to prophylactic treatment having previously failed 4 or more classes of medications (i.e. beta [β]-blocker, anticonvulsant, tricyclic antidepressant, and calcium channel blocker) and behavioural therapy.

**Table 1:** Inclusion and exclusion criteria

Inclusion criteria	Exclusion criteria
• Chronic refractory headache disorder according to *ICHD-3* beta criteria • Age ≥18 years • Migraine attacks that are refractory to medical or behavioural therapy • Eligible for nVNS therapy • Willingness to comply with a defined follow-up interval • Intracranial and cervical pathologies ruled out by an MRI scan • Stable pain medication for 4 weeks prior to nVNS	• Other concomitant neuropsychiatric comorbidity that is not adequately classified or requires a specific diagnosis or treatment • Pregnancy • Previous invasive or non-invasive neuromodulation therapy or ablative procedure • Unwillingness to complete pain diary and provide information on pain intensity and migraine attack frequency (i.e. number of migraine attacks per month) • Cerebrovascular or cardiovascular disease

Abbreviations: *ICHD-3 International Classification of Headache Disorders (3rd edition), MRI* magnetic resonance imaging, *nVNS* non-invasive vagus nerve stimulation

### Stimulation paradigm

The nVNS device (provided by electroCore, LLC, Basking Ridge, NJ, USA) is a handheld, portable appliance that employs a constant voltage-driven signal consisting of a 1-millisecond burst of 5-kHz sine waves repeated at a frequency of 25 Hz, with stimulation intensity ranging from 0 to 24 V. The device is positioned against the side of the neck below the mandibular angle, medial to the sternocleidomastoid muscle and lateral to the larynx. Stimulations are delivered transcutaneously in the region of the cervical branch of the vagus nerve through 2 stainless steel disc electrodes that are manually coated with a conductive gel.

**Fig. 1:**
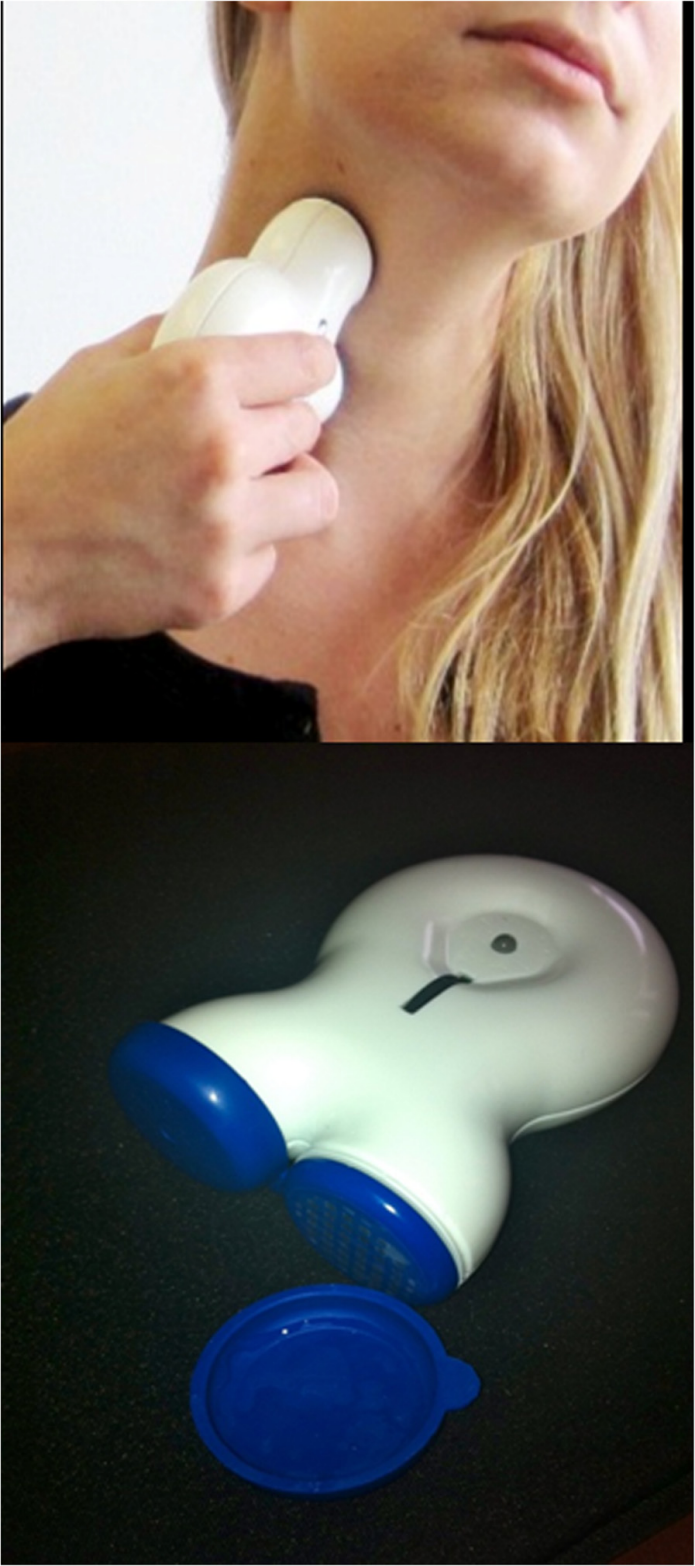
Non-invasive vagus nerve stimulation device

For prophylactic therapy, patients were instructed to administer two 2-min stimulations of nVNS (1 stimulation on each side of the neck in the regions of the right and left cervical vagus nerves) twice daily (morning and late afternoon; total of 4 doses per day) (Fig. 1). For acute therapy, patients were advised to administer two 2-min stimulations (1 stimulation on each side of the neck) at the time of acute medication intake. Before study commencement, all patients received training from the same instructor regarding how to use the device.

### Assessments and end points

Data for all efficacy and safety outcomes were obtained from patient-completed headache diaries and by physician questioning during outpatient visits. Efficacy related to prophylactic therapy was assessed by evaluating the change from baseline in patient-reported pain intensity, number of headache days per month, and number of migraine attacks per month. Baseline values for the number of headache days per month and number of migraine attacks per month were determined on the basis of patient reporting and medical history. The efficacy of acute nVNS treatment on individual attacks was assessed using subjective patient reports of overall pain relief or pain freedom as self-reported at baseline and after 3 months of therapy. Data for all reported and treated attacks were pooled and analysed. The onset, severity, and frequency of treatment-related adverse events (AEs) were evaluated. Furthermore, impaired sleep quality (Pittsburgh Sleep Quality Index [PSQI] score) [30], depressive symptoms (Beck Depression Inventory® [BDI; Psychological Corporation of San Antonio, San Antonio, TX, USA] score) [31], and migraine disability (Migraine Disability Assessment [MIDAS]) scores and grades [32] were evaluated at baseline and after 3 months of nVNS treatment. Sleep disturbances were classified according to the onset of the migraine attack (SM vs NSM) and in relation to the time of sleep evaluation (interictal: 48 h from last attack; or preictal or postictal: <48 h from last attack) (Table 3).

**Table 2:** Demographic and baseline characteristics

Patient No.	Sex	Age, years	Migraine Type	Number of Attacks per Month	Pain Intensity (VAS) Score	Number of Headache Days per Month	MIDAS Grade (score)	BDI Score	Prophylactic Medications	Acute Medications + Time to Achieve Pain Relief	BMI, kg/m^2^	Global PSQI Score
1	f	36	EM+	6	6	10	III (18)	18	4 Classes + Mg + Botox®	Triptan + 60 min	24	5
2	f	66	CM-	6	8	20	IV (45)	19	4 Classes + Botox	NSAID + 90 min	23	12
3	f	41	CM-	7	8	20	IV (39)	34	4 Classes	Triptan + 60 min	31	12
4	m	70	CM-	7	7	15	IV (39)	15	Calcium channel blocker	NSAID + 45 min	25	2
5	f	56	CM+	17	8	18	IV (51)	17	N/A	Triptan + 60 min	25	17
6	f	60	CM-	8	8	22	IV (44)	16	4 Classes + Botox	Triptan + 60 min	24	7
7	f	35	EM-	4	8	9	III (19)	8	4 Classes + Botox	Triptan + 60 min	17	8
8	m	50	EM-	5	7	9	IV (37)	23	4 Classes + Botox	NSAID + 160 min	40	6
9	f	50	EM-	3	9	9	IV (45)	5	4 Classes + Botox	Triptan + 30 min	22	7
10	f	35	CM-	12	8	15	IV (36)	11	4 Classes + Botox	Triptan + 60 min	22	12
11	m	39	EM-	4	8	12	III (19)	21	Calcium channel blocker + Mg + Ca^++^	NSAID + 150 min	29	7
12	f	52	EM-	4	8	12	III (19)	21	4 Classes + Mg	Triptan + 150 min	23	3
13	f	45	CM-	15	8	18	IV (33)	25	4 Classes + Botox	NSAID + 45 min	23	17
14	f	54	EM-	4	8	12	III (18)	17	4 Classes + Mg	Triptan + 120 min	25	4
15	f	60	EM-	10	8	14	III (19)	18	4 Classes + Mg	Triptan + 60 min	18	5
16	f	60	EM-	5	7	14	III (16)	16	4 Classes + Botox	Triptan + 120 min	20	11
17	f	59	CM+	10	8	15	III (18)	16	4 Classes + Botox	Triptan + 90 min	22	6
18	m	72	CM-	9	8	18	III (17)	15	4 Classes	NSAID + 90 min	22	8
19	f	60	CM-	6	8	20	IV (44)	16	4 Classes + Botox	Triptan + 60 min	24	6
20	f	62	EM-	4	7	12	III (16)	15	4 Classes + Botox	Triptan + 120 min	20	10

Abbreviations: *BDI* Beck Depression Inventory, *BMI* body mass index, *Ca* ^*++*^ calcium, *CM* chronic migraine, *EM* episodic migraine, *f/m* female/male, *Mg* magnesium, *MIDAS* Migraine Disability Assessment, *NSAID* nonsteroidal anti-inflammatory drug, *PSQI* Pittsburgh Sleep Quality Index, *VAS* visual analogue scale, *4 Classes* β-blocker, anticonvulsant, tricyclic antidepressant, calcium channel blocker, *−/+* without aura/with aura

**Table 3:** Distribution of sleep patterns

Patient No.	Sex	Age, years	Migraine Type	Migraine Attack Onset	Sleep Evaluation	BMI, kg/m^2^	Global PSQI Score
1	f	36	EM+	NSM	IC	24	5
2	f	66	CM-	NSM	IC	23	12
3	f	41	CM-	NSM	IC	31	12
4	m	70	CM-	NSM	IC	25	2
5	f	56	CM+	NSM	PC	25	17
6	f	60	CM-	NSM	PC	24	7
7	f	35	EM-	NSM	IC	17	8
8	m	50	EM-	NSM	IC	40	6
9	f	50	EM-	NSM	IC	22	7
10	f	35	CM-	NSM	IC	22	12
11	m	39	EM-	NSM	IC	29	7
12	f	52	EM-	SM	IC	23	3
13	f	45	CM-	NSM	IC	23	17
14	f	54	EM-	NSM	IC	25	4
15	f	60	EM-	NSM	IC	18	5
16	f	60	EM-	NSM	IC	20	11
17	f	59	CM+	NSM	IC	22	6
18	m	72	CM-	NSM	IC	22	8
19	f	60	CM-	SM	IC	24	6
20	f	62	EM-	NSM	PC	20	10

Abbreviations: *BMI* body mass index, *CM* chronic migraine, *EM* episodic migraine, *f/m* female/male, *IC* interictal, *NSM* non-sleep migraine, *PC* preictal or postictal, *PSQI* Pittsburgh Sleep Quality Index, *SM* sleep migraine, *−/+* without aura/with aura

### Statistical analysis

Univariate analyses of data obtained at baseline and after 3 months of nVNS treatment were performed to determine changes in pain intensity (visual analogue scale [VAS] score), headache days/month, MIDAS score/grade, number of migraine attacks per month, and depressive (BDI) and sleep (PSQI) comorbidities. Comparisons of the outcomes at baseline and 3 months’ follow-up were performed using the McNemar test for binominal variables and the Student *t* test or the Wilcoxon signed rank test, as appropriate, for continuous data. *P*-values <0.05 were considered significant. All patients were included in the analyses; subgroup analyses were performed for patients with EM and CM. Statistical analyses were performed independently by North American Science Associates Inc. (Minneapolis, MN, USA) using SAS® 9.2 (SAS Institute Inc., Cary, NC, USA).

## Results

### Demographic and baseline characteristics

Of the 20 participants, 16 were female and 4 were male, with an average age of 53.1 years (range, 35–72 years). Ten patients each had been diagnosed with EM (9 without aura/1 with aura) and CM (8 without aura/2 with aura). All patients were classified as MIDAS grade III/IV, with most having clinical signs of sleep disturbance (average PSQI score, 8.3 points; range, 2–17 points) and depressive symptoms (average BDI score, 17.3 points; range, 5–34 points) (Table 2). Evaluation of sleep patterns at baseline revealed that of 20 patients, 15 (6 EM/9 CM) had a disturbed sleep architecture (i.e. global PSQI score of >5 points), and most patients (18/20) had NSM (Table 3). The majority of patients (18/20) had body mass index (BMI) values ≤30 kg/m^2^; 2 patients (1 EM/1 CM) had BMI values >30 kg/m^2^.

### Preventive use of nVNS

**Fig. 2:**
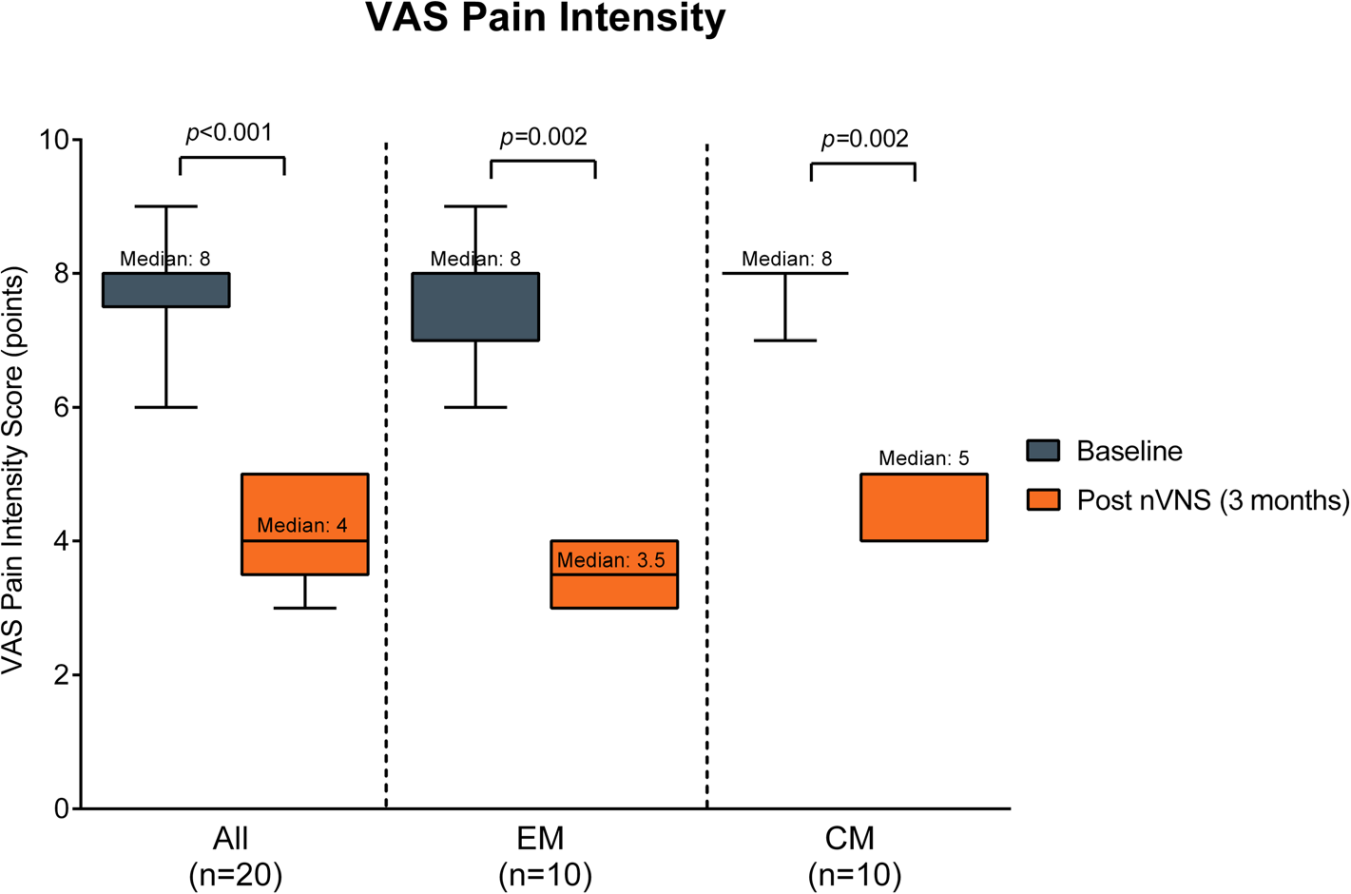
Pain intensity (median [IQR]). Abbreviations: *CM* chronic migraine, *EM* episodic migraine, *IQR* interquartile range, *nVNS* non-invasive vagus nerve stimulation, *VAS* visual analogue scale. Pain intensity (VAS) scores at baseline (blue bars) and after 3 months of treatment with nVNS (orange bars) in all patients, patients with EM, and patients with CM. Data are shown as median (IQR); whiskers indicate the minimum and maximum values

**Fig. 3:**
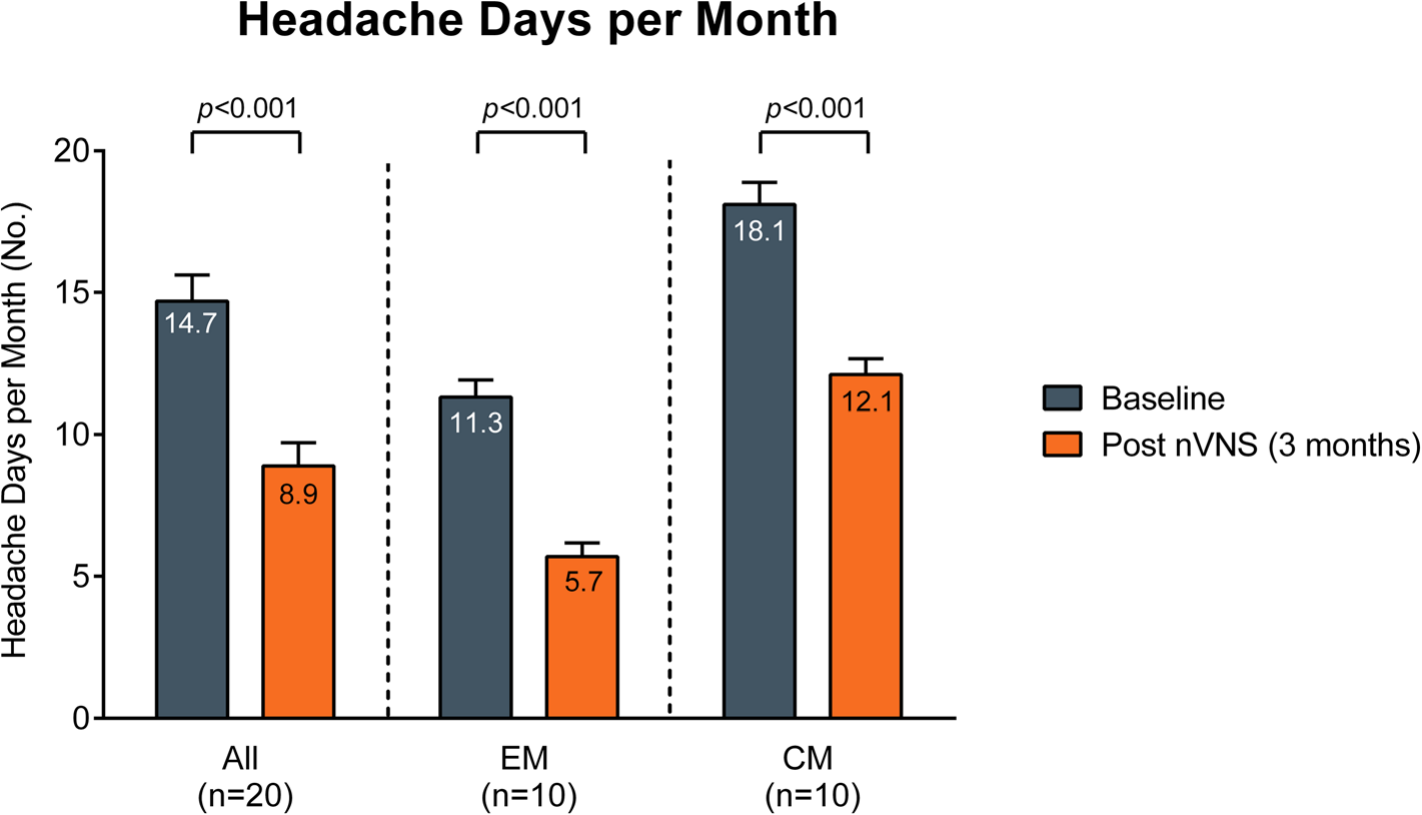
Number of headache days per month (mean ± SEM). Abbreviations: *CM* chronic migraine, *EM* episodic migraine, *nVNS* non-invasive vagus nerve stimulation, *SEM* standard error of the mean. Number of headache days per month at baseline (blue bars) and after 3 months of treatment with nVNS (orange bars) in all patients, patients with EM, and patients with CM. Baseline measures were determined on the basis of patient reporting and medical history. Data are shown as mean ± SEM

**Fig. 4:**
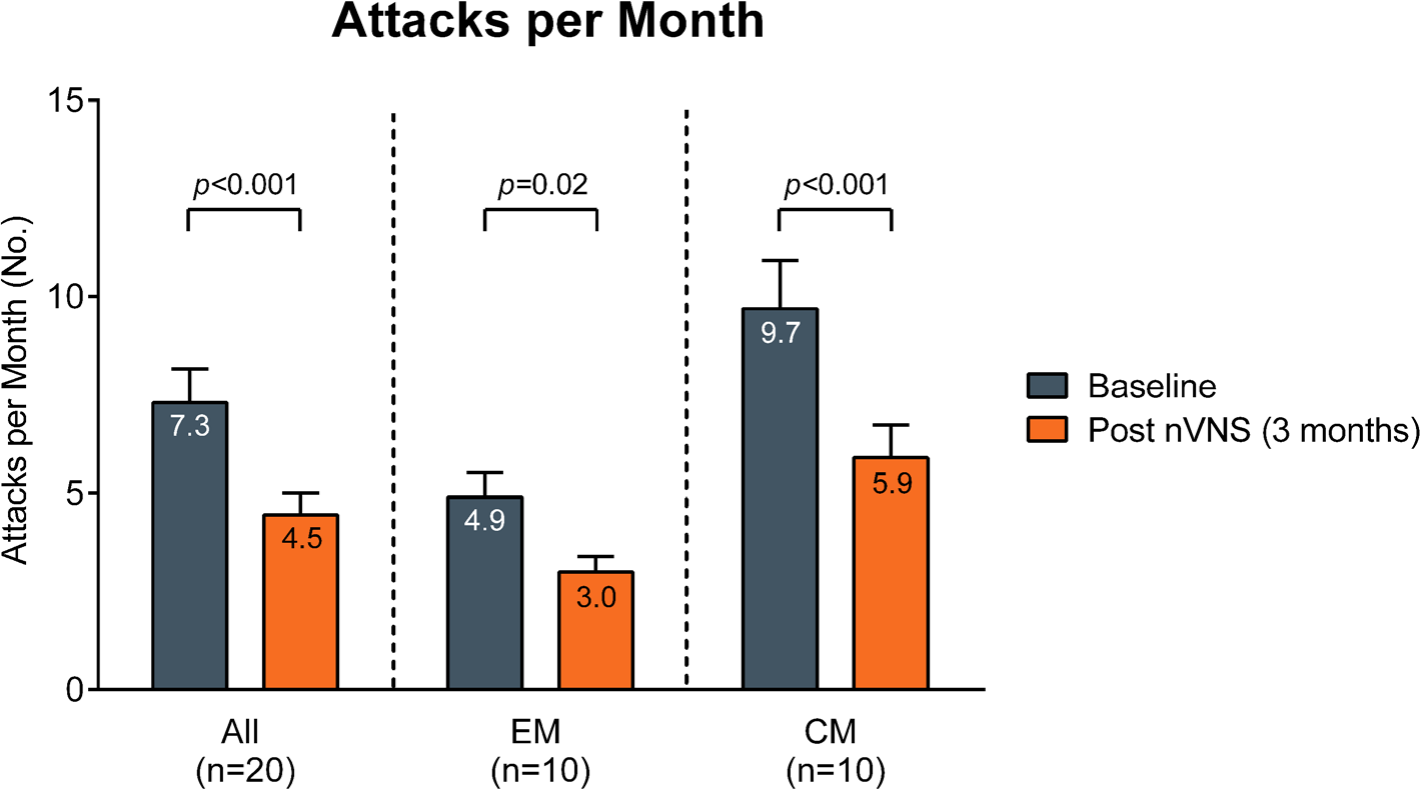
Number of migraine attacks per month (mean ± SEM). Abbreviations: *CM* chronic migraine, *EM* episodic migraine, *nVNS* non-invasive vagus nerve stimulation, *SEM* standard error of the mean. The number of migraine attacks per month at baseline (blue bars) and after 3 months of treatment with nVNS (orange bars) in all patients, patients with EM, and patients with CM. Baseline measures were determined on the basis of patient reporting and medical history. Data are shown as mean ± SEM

Median (interquartile range [IQR]) pain intensity (VAS score) in the total study population was 8 (7.5, 8.0) points at baseline and significantly declined to 4 (3.5, 5) points after 3 months of nVNS use (*p* < 0.001) (Fig. 2). Reductions in pain intensity were observed in both EM (baseline vs 3 months of nVNS treatment: 8 [7, 8] vs 3.5 [3, 4] points; *p* = 0.002) and CM (8 [8, 8] vs 5 [4, 5] points; *p* = 0.002) subgroups (Fig. 2). The overall mean ± standard error of the mean (SEM) number of headache days per month declined from 14.7 ± 0.9 to 8.9 ± 0.8 days (*p* < 0.001) (Fig. 3). Similarly, the number of headache days declined in both the EM (11.3 ± 0.6 vs 5.7 ± 0.5 days; *p* < 0.001) and CM (18.1 ± 0.8 vs 12.1 ± 0.6 days; *p* < 0.001) subgroups (Fig. 3). The number of migraine attacks per month declined significantly in the total population (7.3 ± 0.9 vs 4.5 ± 0.6 attacks; *p* < 0.001), the EM subgroup (4.9 ± 0.6 vs 3.0 ± 0.4 attacks; *p* = 0.02), and the CM subgroup (9.7 ± 1.2 vs 5.9 ± 0.8 attacks; *p* < 0.001) (Fig. 4).

### Acute treatment with nVNS

All patients reported that they had treated each of their migraine attacks with adjunctive nVNS during the 3-month treatment phase. Overall, patients with EM treated 90 migraine attacks with nVNS, and patients with CM treated 177 migraine attacks with the device. All patients self-reported at least some overall pain relief with their pre-existing acute treatment at baseline and with adjunctive acute nVNS use at follow-up. Of the 9 patients who reported a maximum benefit of pain relief at baseline, 5 (2 EM/3 CM) were able to achieve pain freedom within 2 h after initiating adjunctive nVNS treatment as reported at 3 months (*p* = 0.03).

### Migraine-associated comorbidities

**Fig. 5:**
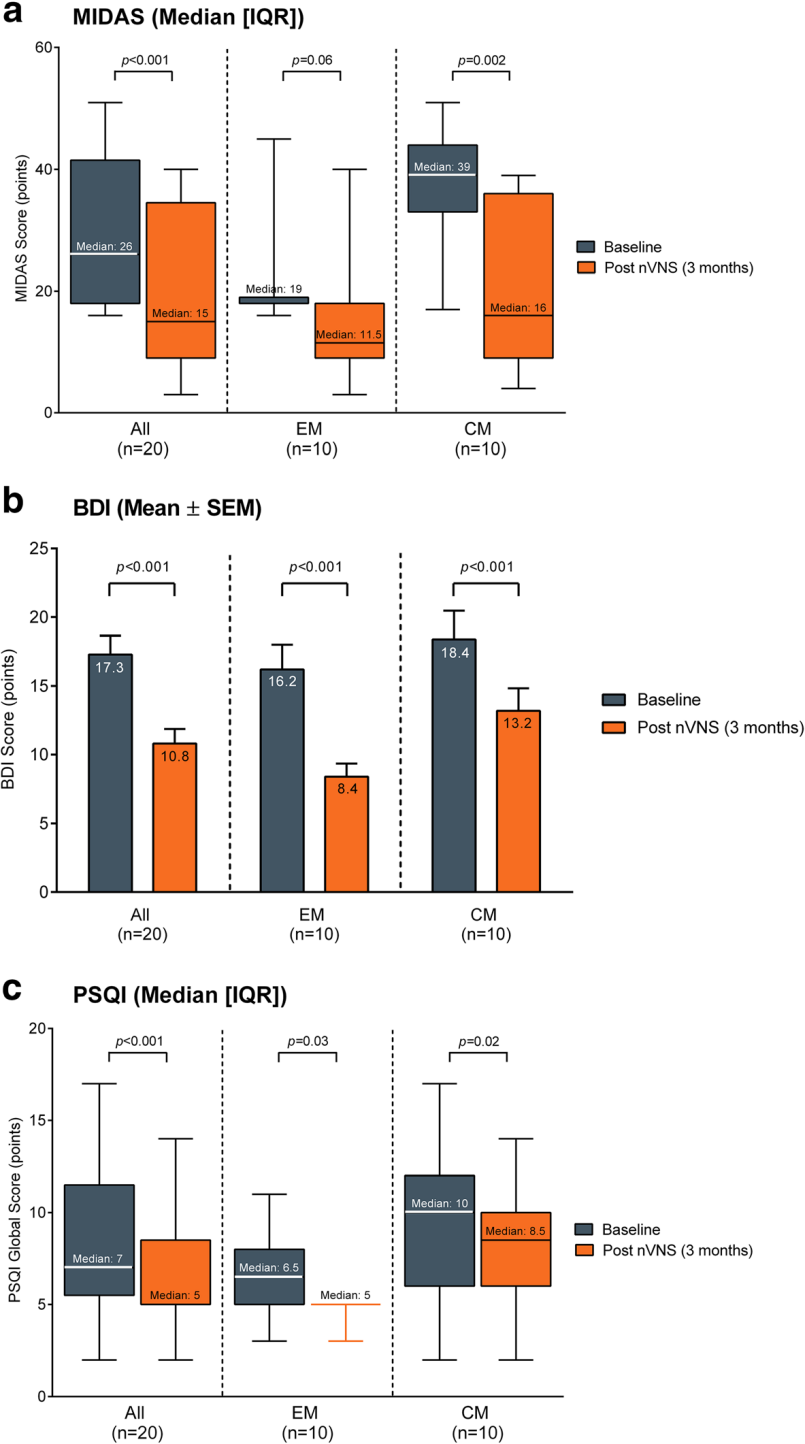
Migraine disability and migraine-associated comorbidities. Abbreviations: *BDI* Beck Depression Inventory, *CM* chronic migraine, *EM* episodic migraine, *IQR* interquartile range, *MIDAS* Migraine Disability Assessment, *nVNS* non-invasive vagus nerve stimulation, *PSQI* Pittsburgh Sleep Quality Index, *SEM* standard error of the mean. MIDAS (**a**), BDI (**b**), and PSQI (**c**) scores at baseline (blue bars) and after 3 months of nVNS treatment (orange bars) in all patients, patients with EM, and patients with CM. Data are shown as median (IQR) for MIDAS and PSQI (whiskers indicate the minimum and maximum values) and as mean ± SEM for BDI

In the total population, significant reductions were observed in the median (IQR) MIDAS score (baseline vs 3 months of nVNS treatment: 26 [18, 41.5] vs 15 [9, 34.5] points; *p* < 0.001) and MIDAS grade (3.5 [3, 4] vs 3 [2, 4]; *p* = 0.008) (Fig. 5a). A significant decrease in the MIDAS score was also observed in the CM subgroup (39 [33, 44] vs 16 [9, 36] points; *p* = 0.002) but not in the EM subgroup (Fig. 5a).

A significant reduction from baseline to 3 months in the mean ± SEM BDI score was noted in the total population (17.3 ± 1.4 vs 10.8 ± 1.1 points; *p* < 0.001), the EM subgroup (16.2 ± 1.8 vs 8.4 ± 0.9 points; *p* < 0.001), and the CM subgroup (18.4 ± 2.1 vs 13.2 ± 1.6 points; *p* < 0.001) (Fig. 5b).

Similarly, significant reductions in the global PSQI score were observed in the total population (7 [5.5, 11.5] vs 5 [5, 8.5] points; *p* < 0.001), the EM subgroup (6.5 [5, 8] vs 5 [5, 5] points; *p* = 0.03), and the CM subgroup (10 [6, 12] vs 8.5 [6, 10] points; *p* = 0.02) (Fig. 5c and Table 4). Reductions in PSQI subscores were significant in the total population for latency (1 [0.5, 3.5] vs 1 [0.5, 2] point; *p* = 0.03) and daytime dysfunction (2.5 [2, 4] vs 2 [1, 2] points; *p* = 0.004) (Table 4). Trends toward lower PSQI subscores after treatment were observed for daytime dysfunction in the EM subgroup (2.5 [2, 4] vs 2 [1, 2] points; *p* = 0.06) and for latency in the CM subgroup (2.5 [1, 5] vs 2 [1, 3] points; *p* = 0.06).

**Table 4:** Pittsburgh Sleep Quality Index (PSQI) scores

	Baseline	Post nVNS	*P-*value
PSQI global score	7 (5.5, 11.5)	5 (5, 8.5)	<0.001
PSQI subscores			
Subjective sleep quality	1 (1, 1)	1 (1, 1)	0.25
Sleep latency	1 (0.5, 3.5)	1 (0.5, 2)	0.03
Sleep duration	0 (0, 0)	0 (0, 0)	N/A
Sleep efficacy	0 (0, 0.5)	0 (0, 0.5)	N/A
Sleep disturbance	2 (1, 2)	1 (1, 2)	0.13
Sleep medication	0 (0, 1)	0 (0, 0.5)	0.13
Daytime dysfunction	2.5 (2, 4)	2 (1, 2)	0.004

Abbreviations: *IQR* interquartile range, *N/A* not applicable, *nVNS* non-invasive vagus nerve stimulation

Data are presented as median (IQR)

### Incidence of AEs

Four patients reported mild treatment-related AEs, most commonly neck twitching and skin irritation. These AEs were transient, coincided with the period of stimulation, and resolved during the course of treatment. No severe or serious AEs occurred.

## Discussion

In this study, a clinically meaningful response to 3 months of prophylactic nVNS therapy was observed in the overall population as well as in the migraine subgroups (EM and CM), and nVNS was associated with significant reductions in pain intensity and number of headache days per month. A significant decrease in the number of migraine attacks per month was noted in the total population and in both subgroups. After 3 months, MIDAS scores and MIDAS grades significantly decreased in the total population. Significant improvements in BDI and PSQI scores were observed for the total population and for both subgroups. Treatment with nVNS was well tolerated with no serious or severe treatment-related AEs.

Migraine is associated with a considerable economic burden [3], and findings from the International Burden of Migraine Study [33] suggest that therapies aimed at decreasing headache frequency and/or headache-related disability are important in containing costs and reducing the clinical and economic strain of migraine. The significant decreases in the number of headache attacks per month and MIDAS scores that were noted in the current study suggest that nVNS may have substantial utility in this regard.

The limitations of the current study include its open-label design, lack of control arm and prospective run-in period, self-recollected reporting of acute pain relief and pain freedom findings, and its small patient population. The lack of a control arm did not allow for examination of the placebo effect, which has been noted consistently in studies of migraine interventions [25, 34]. The method used for reporting acute pain relief and pain freedom was based on patients’ general impressions and did not involve the use of a validated pain scale.

To date, 2 studies have investigated prophylactic therapy for migraine using non-invasive neuromodulation devices [35, 36]. Findings from the current study are consistent with those reported in a study of prophylactic therapy for CM using non-invasive transcutaneous auricular VNS [35] and those reported in a study of prophylactic therapy for migraine using non-invasive transcutaneous supraorbital stimulation [36]. However, unlike the current study, the aforementioned studies did not evaluate the effect of prophylactic nVNS therapy in both EM and CM subgroups.

This study was the first to examine the effect of nVNS on sleep quality in patients with migraine. Significant improvements in sleep quality after 3 months of treatment with nVNS were observed; however, further studies are required to validate these findings. Results from the current study also confirmed the favourable safety profile of nVNS that was reported in previous studies of nVNS in the treatment of migraine [25, 26]. As first-line pharmacologic therapy for the acute treatment of migraine, triptans (i.e. serotonin 5-HT_1B/1D_ agonists) are associated with a risk for cardiovascular/cerebrovascular side effects [37–39]. Thus, nVNS may serve as a safe alternative to triptans, which may potentially lower the risk for medication-overuse headache [40].

The therapeutic effects of nVNS reported in the current study are supported by findings from human neuroimaging studies and from animal studies of migraine pain and cortical spreading depression (CSD) [21, 41–43]. Functional magnetic resonance imaging studies in patients with migraine reflect heightened sensory facilitation, decreased inhibition in response to sensory stimuli, and lack of or decreased adaptation to interictal stimuli [44]. Neuroimaging studies of VNS demonstrate thalamic involvement, which is responsible for processing somatosensory information and regulating cortical activity [41]. Thus, in patients with migraine, nVNS therapy may help to counteract the decline in thalamocortical activity that is responsible for the decreased habituation to interictal stimuli [45]. The potential role of nVNS in migraine-associated pain and CSD has been investigated in animal studies [21, 42, 43]. In a rat model of trigeminal allodynia, Oshinsky and colleagues demonstrated that nVNS decreased trigeminal nociceptive stimulation by inhibiting nitric oxide–induced increases in glutamate levels in the TNC [21]. Further evaluation of the analgesic effect of VNS suggests that VNS inhibits the increase in *c-fos* expression in the TNC that occurs in response to painful stimuli [43]. With regard to migraine aura, which is believed to result from CSD [46], Chen and colleagues compared the effect of direct VNS using implanted VNS with the effect of nVNS in a rat model of CSD [42]. Compared with control treatment, both modes of VNS suppressed CSD susceptibility, with nVNS being more effective than direct VNS [42].

Evidence suggests that the degree of response to nVNS may vary depending on the side of stimulation [47]. Examination of cervical vagus nerve morphology at the site of electrode implantation shows that the right vagus nerve has a considerably larger surface area and more tyrosine hydroxylase–positive nerve fibres than the left vagus nerve, which may be relevant with respect to the side of stimulation [47]. Stimulation-mediated sympathetic- and catecholamine-driven effects and variations in the amount of epineurial connective tissue may modulate treatment response [47]. In the current study, patients administered treatment to both the right and left vagus nerves. However, in 3 recently published studies of nVNS for migraine and cluster headache, stimulation in the region of the right vagus nerve was implemented [25, 26, 29].

## Conclusion

In conclusion, this study demonstrated that administration of nVNS therapy in patients with treatment-refractory migraine was associated with significant reductions in the monthly number of headache days, a preferred outcome measure in migraine studies, and pain intensity. In addition, there were clinically meaningful improvements in migraine-associated disability, depression, and sleep quality. The role of nVNS in migraine therapy is being further explored in ongoing large-scale, randomised, sham-controlled trials with long-term follow-up.

## Abbreviations

AE: Adverse event
BDI: Beck Depression Inventory
BMI: Body mass index
CM: Chronic migraine
CSD: Cortical spreading depression
EM: Episodic migraine
IQR: Interquartile range
MIDAS: Migraine Disability Assessment
NSM: Non-sleep migraine
nVNS: Non-invasive vagus nerve stimulation
PSQI: Pittsburgh Sleep Quality Index
SEM: Standard error of the mean
SM: Sleep migraine
TNC: Trigeminal nucleus caudalis
VAS: Visual analogue scale
VNS: Vagus nerve stimulation
